# Empowering Green Development: How Social Media Interaction Influences Environmental Information Disclosure of High-Polluting Firms

**DOI:** 10.3390/ijerph191610315

**Published:** 2022-08-19

**Authors:** Wei Deng, Jing Shao

**Affiliations:** School of Management, Northwestern Polytechnical University, Xi’an 710072, China

**Keywords:** social media interaction, environmental information disclosure, state ownership, analyst coverage, CEO duality

## Abstract

While social media has become increasingly prevalent in recent years, few studies have examined the role of social media in regulating the environmental information disclosure (EID) of high-polluting enterprises. Using a sample of 2, 211 A-share listed firms in China from 2010 to 2019, this study empirically tests the relationship between firm–investor social media interactions and the EID of high-polluting firms. The results show that social media interaction not only relieves information asymmetry in the capital market, but also triggers market and regulatory pressure for management, ultimately contributing to high-quality EID. The results are robust to a series of alternative estimation approaches and alternative measurements of core variables. Moreover, we found that the positive effect of social media interaction on EID is stronger for enterprises that receive a high level of analyst coverage and for state-owned enterprises (SOEs), but weaker for enterprises whose CEO holds a chairman position (i.e., CEO duality). In addition, further testing shows that social media interaction promotes hard EID to a larger extent than soft information, and the promotion effect is more pronounced for environment-related posts. This study deepens our understanding of how social media supplements formal regulations in the supervision of corporate EID behavior and offers important practical implications for prompting enterprises to achieve high-quality green development.

## 1. Introduction

The problem of the deterioration of the ecological environment hinders the sustainable development of the economy and society. The regulatory authorities regard environmental information disclosure (EID) as a governance method to compel firms to fulfill their environmental responsibilities and exhibit environmentally friendly behavior [1,2]. The listed firms in high-polluting industries are considered the biggest perpetrators of natural environmental pollution and are subject to rigorous scrutiny and high expectations in terms of EID behavior [3,4]. However, many firms comply symbolically with low-quality reports, or even opportunistically use EID to overstate their actual environmental performance [5,6]. Thus, it is imperative to find ways to prompt high-polluting firms to conduct substantive environmental protection behavior and engage in high-quality EID. 

Governments have actively implemented a wide spectrum of formal environmental regulations and policies to urge firms to disclose their environmental information. Command-oriented regulation highlights the regulatory pressure from the government sector, including political embeddedness [7,8,9] and legal regulation [10,11]. Meanwhile, market-based regulations emphasize the effect of economic incentive means, such as environmental tax [12], emission charges [13,14,15], and environmental subsidies [16]. However, few studies have explored how informal regulations such as social media influence corporate EID behavior. In particular, recent studies have been appealing for a greater focus on the role of social media in corporate environmental governance [17], rather than passively responding to external pressure or fitting the existing requirements.

Despite the academic attention directed towards the relationship between social media and corporate voluntary information, the empirical findings remain limited and controversial. Some scholars find that social media plays an active supervisory role in constraining corporate violation disclosure and enhancing the quality of information disclosure [18,19,20]. Lei et al. [19] demonstrate that Twitter-adopting firms are more active in releasing more transparent voluntary nonfinancial information. Lyon and Montgomery [21] suggest that social media increases the information supply to a variety of stakeholders and enables external stakeholders to detect and penalize enterprises for corporate greenwashing behavior. Other scholars reveal opposite evidence, contending social media has a negative effect on voluntary information disclosure [22]. Intense social media attention exposes corporate management to tremendous pressure and highlights expectations, thus compelling firms to engage in low-quality symbolic disclosure to depict an environmentally friendly image and maintain legitimacy [23,24]. Therefore, the relationship between social media and corporate voluntary information disclosure is highly important and controversial. Additionally, the existing literature lacks studies that focus on the impact of social media on corporate EID. Thus, this paper investigates how firm–investor social media interactions may affect the EID behavior of high-polluting enterprises.

China offers a unique and interesting setting to investigate this issue. First, the lack of definite disclosure standards and weak enforcement information disclosure regulations leaves Chinese firms with a high level of discretion to determine what to disclose in environmental reports and how [6,18]. The quality and credibility of corporate EID in China have been subject to severe criticism [5,6]. A new force is required to supervise corporate EID behavior beyond formal regulations. Second, retail investors are the main participants in the Chinese capital market. According to the statistics of the China Securities Depository and Clearing Corporation (CSDC), small investors account for almost 99.77% of the market participants in China. Thus, they play a vital role in supervising corporate behavior. Third, on 1 January 2010, the Shenzhen Stock Exchange released the first interactive platform in China, called “Hudongyi”, (Available online: http://irm.cninfo.com.cn (accessed on 1 March 2022)) to facilitate a two-way interaction between listed companies and investors. Shortly after, the Shanghai Stock Exchange (SSE) introduced a similar online platform, named “easy interaction” (Available online: http://sns.sseinfo.com (accessed on 1 March 2022)). These two novel official social media platforms offer an ideal opportunity for small investors to participate in corporate decisions through asking questions and requesting replies from corporate management.

Based on a sample of heavy-polluting Chinese firms listed in the Shenzhen and Shanghai Stock Exchanges from 2010 to 2019, we first explore how social media interaction impacts the EID quality of high-polluting enterprises. The results show that social media interaction indeed promotes the EID quality of high-polluting enterprises, which supports the monitoring role of social media. This finding is robust to a variety of alternative estimation approaches and alternate measures of core variables. In addition, we examine the mechanism through which social media influences EID from the three perspectives of information function, regulatory intervention, and market pressure. The empirical results show that the positive relationship between social media and EID is more significant for enterprises with high analyst coverage and for state-owned enterprises (SOEs). However, the positive relationship is weaker for firms whose CEOs hold chairman positions (i.e., CEO duality). In a supplementary analysis, we also analyze the heterogeneous effects of social media on EID types and the topic of social media interaction. We find that social media interaction promotes hard EID to a larger extent than soft information disclosure, and the effect of environment-related posts is more pronounced than non-environment-related posts.

This study contributes to the existing literature in the following three ways. First, this study enriches research on the determinants of corporate EID in the Chinese context. Most prior studies shed considerable light on formal institutions, such as command-oriented regulations [10,11,25] or market-based regulations [12,13,14], but ignore the influence of informal regulations, such as social media interaction. This study introduces social media as a supplement for formal regulation in supervising EID activities through facilitating the expression of individual investors’ voices and empowering individual investors to participate in the supervision of corporate EID behavior. Thus, we identify a new method of corporate environmental governance and respond to the call for a more in-depth investigation of cross-disciplinary research on social media and corporate environmental governance [26,27]. 

Second, this paper extends the literature on the consequences of social media interaction on corporate EID decision making. In contrast to prior research that predominantly focuses on how social media is used by listed firms to disseminate their environmental protection efforts from the information perspective [28,29,30], this paper sheds light on how social media interaction exerts pressure on firms to enhance EID quality from the governance perspective. This research adds empirical evidence that social media is a double-edged sword [17], as it not only benefits firms through accelerating the transfer of desired information to a wider audience, but also mobilizes the enthusiasm of external constituents towards participating in the supervision of corporate behavior. 

Third, this paper contributes to the social media literature by elaborating the mechanism through which social media influences EID decisions. While previous research has provided evidence on the positive impact of social media on corporate non-financial disclosure, it is still not very clear how social media influences corporate EID decisions [18,19,20]. We conduct a mechanism-based analysis to verify the three mechanisms of information function, market pressure, and government intervention, which strengthens our understanding of how social media influences corporate EID decisions. 

## 2. Literature Review and Hypothesis Development

### 2.1. Literature Review

Social media can be defined as “a group of Internet based applications that build on the ideological and technological foundations of Web 2.0, and that allow the creation and exchange of user generated content” [31]. With the characteristics of openness, low cost, and interactivity, social media has fundamentally changed the dynamics of corporate disclosure from a one-way mode of “firm–stakeholders” to multi-way interactive communication [17,32,33]. Information dissemination based on social media has reversed the disadvantages of investors, affected the efficiency of capital markets, and improved environmental information [27]. The existing literature mainly focuses on the information dissemination function of social media and shows that social media plays an active role in improving investors’ capability of information acquisition and interpretation [33], relieving information asymmetry [34] and improving the efficiency of capital markets [35,36].

Recently, an emerging branch of the literature extends the consequences of social media interaction to the field of corporate governance by exploring how social media activities influence corporate decisions and improve corporate governance [37]. As it is capable of capturing the wisdom of the crowd and facilitating the expression of stakeholders’ voices, social media represents a valid medium for mobilizing stakeholder activism, and thus has a significant impact on corporate information disclosure decisions [38,39]. For example, Lyon and Montgomery [21] theorize that social media will constrain the occurrence of corporate greenwashing. Zhou et al. [40] reveal that investors’ social media attention can force firms to take corrective action after the occurrence of a misreport. Nie and Jia [41] show that social media empowers minority shareholders to participate in corporate governance and promotes management earnings forecast accuracy. 

While previous studies have advanced our knowledge, the empirical evidence on whether and how social media interaction impacts corporate non-financial information disclosure, especially EID, is limited. This paper attempts to explore this in depth in the Chinese context, so as to provide a new research perspective and empirical evidence for the interaction between social media and corporate environmental governance.

### 2.2. Hypotheses Development

Legitimacy theory laid the basis for examining how social media changes corporate environment behavior and promotes social change [42,43]. Legitimacy theory posits that firms attempt to enhance or protect their legitimacy, which is defined as “a generalized perception or assumption that the actions of an entity are desirable, proper, or appropriate within some socially constructed system of norms, values, beliefs and definitions”, to ensure the continuous resource support from their stakeholders [44]. Concerns surrounding legitimacy loss forces firms to implement practices to comply with institutional norms and social values and adapt their own behavior to deal with external pressure [45]. Firms in high-polluting industries have legitimacy disadvantages and are prone to becoming victims of social media activities [18,25]. The EID behavior of high-polluting firms has increasingly become an important aspect of legitimacy management behavior [46,47]. 

According to legitimacy theory, we hypothesize that firm–investor social media interaction promotes the EID of high-polluting enterprises through the information function and triggering market and regulatory pressure. First, social media interaction enhances investors’ capability of information acquisition and interpretation, relieves information asymmetry in the capital market, and therefore compels firms to improve the transparency of the EID. Social media platforms, such as the E-interactive platforms initiated by stock exchanges, facilitate direct communication between firms and retail investors, and shorten the distance between participants in the process of information disclosure [48]. Through direct interaction with corporate management, small investors can raise questions and take their information appeal to corporate management in a timely manner at an almost zero marginal cost [37] and ask corporate management to respond. In this sense, the participation of investors in the interaction with the company’s management on social media platforms helps to increase their ability to obtain and interpret information and eliminate their unfavorable information disadvantage, thus mobilizing the enthusiasm of individual investors who otherwise would be marginalized to participate in corporate governance. With the improvement of corporate governance, the agency-related problem of hiding information and management opportunism in EID decisions can be effectively regulated.

Second, social media interaction can focus public attention and stimulate market and regulatory pressure for firms to enhance their EID quality. Aided by the characteristics of openness and convenience [49], social media platforms attract inbound audiences to communicate with firms, which leads to extensive public attention. This intense attention aggregates individual actions into a large-scale collective action [36] and gathers the power of crowds [33,50]. On the one hand, the failure to address online appeal may result in the quick spread of negative publicity and thus damage the firm’s reputation [39], which will eventually be reflected in the decline in the company’s stock prices and trading volume, and even raise the likelihood of the CEOs of the targeted firms being downgraded or dismissed through investors “voting with feet” [51]. Previous studies also provide evidence that aggregated social media attention exerts pressure on firms to improve their information disclosure to preempt activism at their firms and avoid potential risks [52]. On the other hand, heated discussions and massive interactions on social media may introduce a large amount of attention to and scrutiny from the regulatory authorities [20,40], which may increase the risk of administrative intervention and legal punishment.

In summary, firm–investor social media interaction plays an active role in supervising the EID behavior of high-polluting firms through strengthening investors’ information acquisition capabilities and triggering market and regulatory pressure on management. Therefore, we propose the following hypothesis:

**Hypothesis 1 (H1).**  *The firm–investor social media interaction has a positive effect on the EID quality of high-polluting firms.*

## 3. Research Design

### 3.1. Sample Selection and Data Sources

The initial sample included Chinese A-share high-polluting enterprises from 2010 to 2019. This study chose 2010 as the starting year of the sample because the Shenzhen Stock Exchange developed the first online interactive platform, called “Hudongyi”, in 2010. Before that, no official E-interactive platform existed for the direct exchange between investors and listed firms. We identified 16 high-polluting industries (thermal power, steel, cement, electrolytic aluminum, coal, metallurgical, chemical, petrochemical, building materials, papermaking, brewing, pharmaceutical, fermentation, textile, leather, and mining) based on the “Guidelines on environmental information disclosure of the listed companies” issued by the Ministry of Environmental Protection of the People’s Republic of China in 2010.

**Figure 1:**
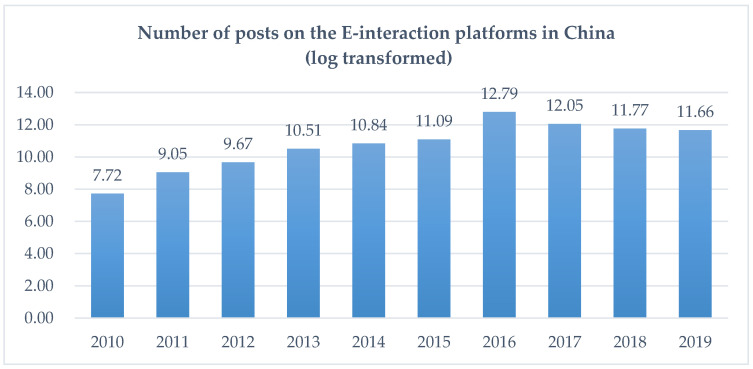
Trends in the volume of posts on the E-interaction platforms (log-transformed) in China.

We used the unique and detailed original posting data on the E-interaction platforms initiated by the Shanghai and Shenzhen Stock Exchange. This context offered a unique opportunity to test our theoretical hypotheses. First, in contrast to other online platforms, such as Twitter, Microblog, and Stock Message Boards, which lack the supervision of official regulation, the E-Interaction platform is built under the supervision of the stock exchange, and its operating environment is more reliable. Firms are required by the stock exchange to check the questions in the E-Interaction platform on time and reply within two days. In this sense, corporate management’s identification of the internet stakeholders’ opinions expressed on this platform is expected, and the latter has a stronger influence on corporate decisions than other social media platforms. Second, this platform establishes a Q and A section, and all the Q and A records are open to all visitors, including usernames, user IDs, and the dates and contents of posts, as well as the firms’ replies. This setup provided the access to the data for our study. As shown in Figure 1, the volume of postings on the E-interaction platforms increased gradually in China from 2010 to 2019.

The data sources of our study are listed as follows: (1) Data on social media interaction were collected from the official E-interactive platforms initiated by the Shanghai and Shenzhen Stock Exchange. We used the Python program to automatically crawl all the detailed original postings data for each firm via the two E-interactive platforms from 2010 to 2019. (2) The information on corporate EID was manually extracted using the content analysis of corporate annual reports or corporate social responsibility reports. (3) Data on corporate ownership, analyst coverage, and basic financial indicators were collected from the China Stock Market and Accounting Research (CSMAR) database, which has been widely used in prior research [4,5,20].

After merging the above datasets, the following steps were taken for the sample selection process: (1) we excluded firms with abnormal financial conditions, such as those defined as ST and *ST companies. (2) We removed observations with at least one missing control variable. Finally, a total of 13,936 firm-year effective observations from 2211 enterprises were obtained.

### 3.2. Variables and Models

#### 3.2.1. Corporate EID

The content analysis method is a mainstream method to measure enterprise EID quality in existing studies. Following prior research [1,2,3], this study assessed the quality of EID through adopting the 10-item list of the environmental information disclosure evaluation system. The ten items include both hard and soft information disclosures. The scoring index consists of the following 10 items: (1) corporate investment in environmental protection and green technology; (2) government subsidies and grants related to environmental protection; (3) pollution emission and reduction; (4) ISO environmental system certification; (5) actions taken to improve the natural environment; (6) the influences of environmental protection policies; (7) loans for environmental protection; (8) litigation, penalties, and awards associated with the environment; (9) goals and visions concerning corporate environmental protection; and (10) other issues of expenditure and income related to the environment. From the above scoring index, items (1), (2), (3), (6), (7), (8), and (10) represent quantitative and monetary information, and are classified as hard information disclosures, whereas (4), (5) and (9) represent qualitative non-monetary information and are classified as soft information disclosures.

For each item, we assigned 0 points in the case of non-disclosure, 1 point for quantitative disclosure, 2 points for non-monetary information disclosure, and 3 points for qualitative monetary information disclosure [47]. Each enterprise obtains an aggregate score by adding the score of each item using the following formula:

$${\mathrm{EID}}_{\mathrm{it}}=\sum_{i=1}^{n}{\mathrm{eid}}_{\mathrm{ijt}},$$

where ${\mathrm{EID}}_{\mathrm{it}}\;$ is the total score of the EID quality for the firm i in the year of t, and ${\mathrm{eid}}_{\mathrm{ijt}}\;$ is the score of the item j for the firm i in the year of t. The variable j covers data between a score of 1 and 10 in the scoring index. A larger value suggests a higher quality of the corporate environmental disclosure.

#### 3.2.2. Social Media Interaction

Following prior studies [39,40,41], we used the number of questions posted on the E-interaction platforms to indicate the level of social media interaction. We first created a continuous variable, INTERACT, calculated via the natural logarithm of one plus the total number of effective questions (i.e., questions with management replies) posted by the individual investors on the E-interactive platforms for each firm. For the robustness test, we also used the natural logarithm of one plus the total number of words of each question for each firm as a proxy for social media interaction. A larger value indicates a stronger firm–investor interaction on social media platforms.

### 3.3. Model Building

To test H1 and examine the relationship between social media interaction and EID quality, this study employs the ordinary least squares (OLS) regression, and the regression model is designed as follows:

(1) $${\mathrm{EID}}_{i,t+1}=\beta_{0}+\beta_{1}{\mathrm{INTERACT}}_{i,t}+\beta_{2}{\mathrm{Control}}_{i,t}+\mathrm{Year}+\;\mathrm{Industry}+\varepsilon_{i,t}$$

Among the above models, ${\;\mathrm{EID}}_{i,t+1}\;$ is the dependent variable, representing the quality of the corporate environmental disclosure of firm i in year t + 1. ${\mathrm{INTERACT}}_{i,t}$ is the number of questions posted on the E-interactive platforms of firm i in year t. $\varepsilon_{i,t}\;$ is the random error. A positive and statistically significant $\beta_{1}$ in model (1) would support H1.

To eliminate potential confounding effects, we included a series of control variables that may influence the impact of social media activism on EID. Referring to the prior literature ([6]), we controlled the firm size (SIZE), which is calculated as the natural logarithm of the total assets of the firm. To control the profitability of polluting firms, we included the ROA, which was measured via the net income divided by the total assets of the firm. The leverage of the firm (LEV) was measured using the total debt over the total assets. We also considered several corporate governance factors. We controlled the size of the board of directors (BOARD), the proportion of independent directors on the board (INDEP), the shareholding ratio of the largest shareholder (TOP1), and the institutional ownership (INS). Moreover, we controlled the regional market development level (MARKET) by using the provincial marketization index developed by Fan et al. [53]. Table 1 presents the definitions of the variables used in this study.

## 4. Empirical Results

### 4.1. Descriptive Statistics

Table 2 presents the description of the main variables. To eliminate the interference of outliers, all the continuous variables were winsorized at the top and bottom 1% levels. The maximum value of the EID quality was 27 with a mean value of 5.071, and the standard deviation was 5.792. These values indicated that the average EID quality of Chinese high-polluting enterprises was generally low, and there was significant variation in the EID quality within the samples. In addition, the minimum, maximum, and mean values of INTERACT were 0, 6.472, and 2.209, respectively, suggesting that the sample firms received an average of 4.25 questions on the E-interactive platform every year, with significant variations across different enterprises. However, some firms received no questions from investors on the social media platform. Furthermore, the mean value of the SOE was 0.573, indicating that state-owned enterprises accounted for 57.3% of all the high-polluting enterprises in China, which is consistent with reality. Finally, the statistics of the other basic financial status indicators are consistent with existing related research results [1,4,18].

### 4.2. Pearson Correlation Analysis

Table 3 presents the Pearson correlation matrix for the main variables. The correlation between EID and INTERACT was 0.371, which was significant at the 5% level. This correlation result suggests a positive relationship between social media interaction and the quality of the EID, which provides preliminary support for H1. Moreover, the quality of the EID was also significantly correlated with the firm size (SIZE), leverage (LEV), the board size (BOARD), institutional shareholding (INS), and state ownership (SOEs). Furthermore, the maximum pairwise correlation between SIZE and between OUTDIR and BOARD INS was 0.436 below the maximum threshold of 0.7, and the maximum VIF values of all the variables were below the threshold of 10, indicating that multicollinearity was not a serious problem.

### 4.3. Main Regression Results

Table 4 presents the results of an OLS regression examining the impact of social media interaction on EID quality. Column (1) includes all the control variables; Column (2) includes the independent variable and all the control variables. Before including the independent variable, the adjusted R^2^ for Column (1) was 10.4%. After adding the independent variable of INTERACT, the explanatory power of the model rose to 12.1%. As shown in Column (2), the coefficient of INTERACT is positive and statistically significant at the 1% level (coefficient = 0.236, t-stat = 10.483), lending support for H1. The above result confirms our expectation that firm–investor social media interaction exerts pressure on firms to disclose high-quality environmental information.

Additionally, in terms of the control variables, we found that enterprise size (SIZE), leverage (LEV), board size (BOARD), and market development (MARKET) were significantly positively associated with EID, indicating that larger enterprises, enterprises with a larger board of directors, and enterprises located in regions with a high level of market development are likely to issue high-quality EIDs.

### 4.4. Robustness Checks

#### 4.4.1. Alternative Measure of Core Variables

First, referring to prior research [54], we used the content scoring (C-score) of the RKS CSR ratings as an alternative measure of the EID quality. We replaced the dependent variable EID with the RKS C-score, and Column (1) of Table 5 presents the regression results. As shown in Column (1), the coefficient remained positive and significant at the 5% level (coefficient = 0.216, *p* < 0.01), which is consistent with our main regression model in Table 4.

Second, following Nie and Jia [41], we also employed the number of words of each question posted on the E-interactive platforms as an alternative measure of social media interaction and reran the regression model using the natural logarithm of one plus the number of words of each question (INTERACT2) as an independent variable, and the results are presented in Column (2) of Table 5. As shown in Column (2), the regression coefficient for INTERACT2 was positive and statistically significant (coefficient = 0.116, *p* < 0.01), which is consistent with the main results, and verifies the robustness of our baseline regression results.

#### 4.4.2. Alternative Estimation Approaches

First, to alleviate the endogenous problems caused by time-invariant unobserved firm heterogeneity, the firm fixed-effect model was adopted. We also conducted Hausman’s tests to confirm whether the fixed- or random-effect models should be used. The results of the Hausman test (*p* < 0.01) rejected the null hypothesis that the random effect model is suitable. Therefore, the fixed effect model was appropriate. Firm dummies were included to control for the firm fixed effect. The fixed-effect model regression results are depicted in Column (1) of Table 6. As show in Column (1), the coefficients of INTERACT were still significantly positive.

Second, since EIDs are non-negative data and a many of the data were zero, we followed Meng et al. [6] to adopt the Tobit regression model as a robustness test to address the censored and truncated data issue. Column (2) of Table 6 reports the Tobit regression results. As shown in Column (2), the coefficient for INTERACT was positive and statistically significant (coefficient = 0.804, *p* < 0.01), which is consistent with the baseline regression results.

Third, to deal with the endogeneity originating from the simultaneity and the dynamic relation between social media interaction and EID, we applied the two-step GMM (Generalized Method of Moments) model to our panel data. The results of the GMM regression model are reported in Column (3) of Table 6. In order to enhance the reliability of the regression results, the rationality of the model setting and the validity of the instruments variables were tested; the AR(1) test was significant and the AR(2) test was insignificant (*p* = 0.746), indicating that there were no autocorrelation problems of the disturbance term. The result of the Hansen test (*p* > 0.5) rejected the possibility of over-identification. The above results show that the GMM regression results are valid. The coefficient for INTERACT was positive and statistically significant (coefficient = 0.238, *p* < 0.01), confirming the robustness of our baseline results.

#### 4.4.3. Addressing the Endogeneity Problem

Another potential concern for the main result is reverse causality. For example, firms exhibiting high-quality EID may attract high levels of online attention on the social media platform. To address such a potential reverse causality, we employed a 2SLS instrumental variable approach. Following prior research [26], we adopted the natural logarithm of the annual provincial internet broadband access port volume (NETZEN) as the instrumental variable, as it may affect the number of participants in the E-interactive platforms but has no direct impact on corporate EID. In the first-stage regression model, we used NETZEN as the instrumental variable. In the second-stage regression model, we deployed the predicted social media activism (PREINTERACT) derived from the first-stage regression model to examine the effect of the predicted social media interaction on EID. The F-statistics of the first-stage model exceeded the threshold of 10, revealing that the selected instruments were not weak. Table 7 presents the results of the instrumental variable approach. The parameter estimates of PREINTERACT in Model (2) was 1.122 and significant at the 1% level, suggesting that the effect of social media activism on EID remains valid after considering the endogenous problem.

## 5. Mechanism Analysis

The above sections provide empirical evidence that firm–investor social media interaction promotes the high-quality EID of heavy-polluting enterprises. This section attempts to further elaborate the three mechanisms through which social media promotes the EID of heavy-polluting enterprises.

### 5.1. Information Effect

Based on the previous argument, the first channel through which social media can promote the EID of high-polluting firms is through the information function of relieving information asymmetry. We assume that social media interaction will have a greater impact on the EID of enterprises that receive more coverage from financial analysts by alleviating the information asymmetry and opportunistic behavior in their EID decision making.

Financial analysts are employed by securities institutions, and they operate completely independently and have no conflict of interests with the target companies. The existing literature shows that financial analysts care about enterprises’ environmental performance, and they integrate enterprises’ environmental and social performance indicators into the evaluation of enterprise value [55]. As critical information intermediaries in the capital market, they can monitor and constrain firms’ misbehaviors by focusing market and public attention on the firms [56,57]. Consequently, firms with a great degree of analyst coverage will be stimulated to reduce opportunistic behavior and provide high quality EID [58]. 

To test the function of financial analyst coverage, this paper introduces the interaction between analyst coverage (ANALYST) and social media interaction (INTERACT). As shown in Column (1) of Table 8, the coefficient for the interaction term of INTERACT*ANALYST was 0.023 and significant at the 5% level, indicating that the impact of social media interaction on firms’ EID is stronger for firms with a high level of analyst coverage.

### 5.2. Market Pressure Effect

The second channel through which social media can promote the EID of high-polluting firms is the management of market pressure. We confirmed the relevance of the market pressure mechanism by examining whether the CEO also holds the chairman position. According to agency theory, when the CEO and the chairperson of the board is the same person (i.e., CEO duality), the effectiveness of board monitoring is reduced [59,60]. Such CEOs care less about market pressure, as they are less likely to be punished by the board when they delay paying attention to investors’ social media opinions [61]. Therefore, such CEOs are less sensitive to assessments from the capital market, and their decisions are less likely to be swayed by the market pressure from capital market participants, such as retail investors. In contrast, for CEOs that do not hold chairman positions, failing to respond to investors’ claims on social media could more easily raise the likelihood of being punished by the board or even being fired.

To test the influence of CEO duality, this paper introduces the interplay of social media interaction and CEO duality (equals 1 if the CEO is also the chairman; otherwise 0). In Table 8, Column (2) shows that the coefficient of the interaction term of INTERACT*DUALITY was −0.139 and significant at the 1% level. This regression result indicates that the promoting effect of social media interaction on EID is weaker for firms whose CEO holds a chairman position (CEO duality) that makes them immune to market pressure.

### 5.3. Regulatory Intervention Effect

The third channel through which social media promotes the EID of high-polluting firms is by triggering regulatory intervention. We verified the regulatory intervention mechanism by examining the moderating effect of state-owned property. Among all the firms, the state-owned enterprises (SOEs) are the most influenced by the government visions and missions [5,18]. In SOEs, even the top executives are appointed by the government and receive instructions from government agencies [62]. Thus, compared with non-SOEs, SOEs will bear the brunt of government pressure and will be more concerned about government intervention, such as government investigation or punishment for a lack of environmental transparency or other noncompliance [18,63].

To examine the impact of state-owned property, we included the interaction term between INTERACT and SOE. As shown in Column (3) of Table 8, the coefficient for the interaction term of INTERACT * SOE was 0.152 and significant at the 1% level, revealing that SOE strengthens the positive relationship between social media interaction and EID quality. Compared with private firms, SOEs are more susceptible to regulatory pressure from social media interaction and are more likely to implement substantial environmental actions and release high-quality EIDs.

## 6. Further Analysis

### 6.1. Considering the Types of EID

Do firms respond to legitimacy pressure triggered by social media with substantive disclosures, namely hard quantitative information in their EIDs, or merely symbolic disclosures that use vague qualitative information to depict an environmentally friendly image? Based on the specific type of environmental disclosure, EID can be divided into two categories: hard and soft information disclosures [1,64]. Compared with hard information disclosure, which is objective and verifiable, soft information disclosure involves symbolic environmental commitment without details or substantiation. The distinction between hard and soft information disclosure is depicted in Section 3.2.1. The subsample regression results are presented in Table 9. As shown, the regression coefficient of INTERACT for hard information disclosure in Column (1) was 0.235 and significant at the 1% level. In contrast, the regression coefficient of INTERACT for soft information disclosure in Column (2) was positive but insignificant. The above results indicate that social media interaction plays a significant monitoring role in prompting substantial and verified hard information as opposed to unverified soft information to manipulate the impressions of stakeholders.

### 6.2. Considering the Topic of Social Media Interaction

In the main regression, we did not discern the specific topic of each question posted by investors on the social media platforms. However, the questions posted on social media platforms covered a variety of topics, including enterprise operations, mergers and acquisitions, personnel changes, and environmental issues. Do different questions have heterogeneous impacts on the EIDs of high-polluting firms? To explore this question, building on Liu et al. [53] and Wang and Jia [65], we adopted a dictionary-based approach to identify the environment-related questions on the E-interactive platform, and employed the Stata program to identify these environment-related questions on the basis of whether or not the questions included any of the established environment-related keywords (e.g., green, nature, environment, and ecology). Then, we divided all the questions posted on the social media platforms into two types: environment-related questions (HJPOST) and non-environment-related questions (NHJPOST). After dropping the samples without investors’ questions, we reran the OLS regression model. The regression results are presented in Table 10. As shown in Column (1), the regression coefficient for HJPOST was positive and statistically significant (coefficient = 0.781, *p* < 0.01). The regression coefficient for NHJPOST was also positive and statistically significant (coefficient = 0.224, *p* < 0.01), and the coefficient difference between the two groups was significant (*p* < 0.05). The above results indicate that both environment-related posts and non-environment-related posts on social media can significantly promote the EID quality of high-polluting enterprises, but the effect of environment-related posts is more pronounced.

## 7. Discussion

### 7.1. Practical Implications

Our research has several important practical implications. First, the government and policymakers should fully recognize the importance of social media supervision in promoting the green transformation of listed firms in this new era of information and take measures to guide enterprises in strengthening the participation of online interactions with investors on social media platforms. According to our results, social media interaction is an effective external supervision force with a practical significance for prompting the high-quality disclosures of heavy-polluting enterprises. Our research demonstrates that stimulating the efficiency of external governance by giving full play to the supervisory role of social media is the key to enhancing the EID quality of highly polluting enterprises. Thus, the government should implement measures, including the formulation of related regulations, to seek firms’ assurance on the reliability of online participation and integrate the online interaction quality into listed companies’ information-disclosure-performance evaluations.

Second, high-polluting firms, especially those owned by the state and receiving intensive analyst coverage, should be fully aware of the challenges brought about by the new media environment and adopt proactive information disclosure strategies. According to the analysis above, social media empowers individual investors to voice their concerns to management directly and to tap into the wisdom of the crowd, which triggers market pressure and regulatory pressure for enterprises and influences corporate EID decisions. Therefore, as the main perpetrators of ecological deterioration, high-polluting firms should respond actively to social media pressure and adopt substantial environmental actions to express their commitment and effort to fulfill sustainable development instead of complying symbolically through unverified information disclosure. Specifically, when targeted by intense social media activism, high-quality EID and increased hard information disclosure are encouraged for firms to cope with external institutional environmental pressure.

Third, analysts—as important information intermediaries—should play a more active role in supervising enterprises’ EID behavior. Analyst coverage can largely alleviate the problem of information asymmetry inside and outside the enterprise and exert pressure on the top executives to restrict window dressing regarding EID behaviors. Our empirical results demonstrate that analyst coverage will aggravate firms’ vulnerability to social media pressure and strengthen firms’ motivation to adapt EID as a way of cultivating legitimacy. Thus, analysts should place more emphasis on the environmental aspects of listed enterprises when they release stock reports or make stock recommendations, especially for those enterprises with poor environmental performance or low EID quality.

### 7.2. Limitations and Future Research Directions

There are some limitations that can be further explored in future research. First, while we take the E-interaction platforms initiated by the stock exchanges as the research scenario, many other new media platforms, such as Guba, Weibo, and WeChat, persistently provide good channels for market participants to exchange information. Despite being useful representatives, E-interaction platforms are unable to fully cover the interactions between firms and their investors. Greater attention should be focused on the influence of these social media platforms. Second, we did not distinguish between the positive or negative emotions of each question on the E-interaction platforms. Instead, we only assumed that the questions posted on the platforms were equally important, which would put pressure on the corporation and would in turn influence corporate EID behavior, regardless of whether their emotions were positive or negative. Future research should further explore the detailed textual features of social media posts, such as emotions and readability. Third, we contextualized this research in China, a developing country where the weak enforcement of information disclosure regulations and the imperfect government regulation system blunt the effectiveness of formal regulations. However, whether the findings are applicable to other developed countries remains to be explored. Future studies should examine our conclusions in developed countries where the formal regulations are strict.

## 8. Conclusions

In this study, we investigated whether and how firm–investor social media interaction influences the EID quality of high-polluting firms in an attempt to clarify the supervisory role of social media interactions. Using panel data on Chinese high-polluting listed firms, we drew the following conclusions:

First, the empirical results show that firm–investor social media interactions can significantly promote the EID quality of high-polluting firms, confirming the supervisory role of social media in promoting substantial information disclosure. The extant literature mainly focused on offline, formal institutions such as command-control regulation [7,8,9,10,11] and market-incentive regulations [12,13,14,15,16]. To our knowledge, this study is one of the very few to investigate the impact of social media interaction on corporate EID. This study demonstrates that social media interaction can relieve information asymmetry between firms and external stakeholders and trigger both market and regulatory pressure for the firms; therefore, it compels firms to implement high quality environmental disclosures. Our results are consistent with those of Fan et al. [18] and Lei et al. [20], verifying that social media supplements formal regulation in empowering environmental activism and responds to the call for a more in-depth investigation of how changes in information technology, such as the application of social media, affect corporate disclosure practices [14,32,36].

Second, this study further reveals that the positive impact of social media interaction on EID is moderated by financial analyst coverage, state-owned property rights, and CEO duality. The results show that the promoting effect of social media interaction on EID is more pronounced for enterprises with a high level of financial analyst coverage and for state-owned enterprises but is weaker for enterprises whose CEOs hold a chairman position. The above results demonstrate that analysts serve as key information intermediaries in the capital market through which social media pressure is transformed into corporate EID decisions [66], and that the impact of social media on corporate EID behavior depends heavily on firms’ property rights and CEO’s discretion power. These findings strengthen our understanding of the underlying mechanism through which social media affects the EID of high-polluting firms.

Third, the heterogeneity analysis shows that social media interaction promotes hard information disclosure to a larger extent than soft information disclosure. This result further confirms the effectiveness of social media in putting pressure on high-polluting enterprises to implement substantial environmental actions and is in accordance with the view in previous studies that enterprises tend to disclose more detailed environmental information and take substantive measures under greater legitimacy pressure [47,67,68]. Moreover, we also consider the topic of social media interaction, and we found that not only environment-related posts, but also non-environment-related posts, can promote the EID of high-polluting enterprises; however, the impact of environment-related posts is more pronounced. This finding echoes the notion in the prior literature [19,40,63] that the attention brought by social media puts pressure on the management and prompts them to behave in a more conservative and honest manner.

## Figures and Tables

**Table 1:** Variable Definitions.

Variables	Definitions
EID	Total score of 10 EID dimensions.
INTERACT	Natural logarithm of 1 plus the number of questions posted on E-interaction platforms.
SIZE	Natural logarithm of total assets.
ROA	Ratio of net income to total assets.
LEV	Ratio of total liabilities to total assets.
BOARD	Natural logarithm of the number of boards of directors.
INDEP	The proportion of independent directors on the board.
TOP1	The shareholding ratio of the first largest shareholder
INDEP	The proportion of independent directors on the board of directors.
MARKET	The relative index of marketization process in each region from Fan et al. (2007) [53].
INS	The shareholding ratio of the institutional shareholder.
SOE	A dummy variable that is equal to 1 if a firm’s ultimate shareholder is the state and is otherwise 0.
ANALYST	Natural logarithm of number of financial analysts that issued earnings forecasts reports for the firm.

**Table 2:** Descriptive statistics.

Variable	N	Mean	SD	Min	Median	Max
EID	13936	5.701	5.792	0.000	4.000	27.000
INTERACT	13936	2.096	2.290	0.000	0.693	6.472
SIZE	13936	21.871	1.229	19.540	21.693	25.700
ROA	13936	0.068	0.065	−0.142	0.061	0.279
LEV	13936	0.400	0.207	0.045	0.388	0.929
BOARD	13936	1.707	0.979	0	2.197	2.773
INDEP	13936	0.372	0.052	0.181	0.333	0.667
TOP1	13936	0.360	0.150	0.091	0.342	0.773
INS	13936	0.374	0.239	0.001	0.380	0.887
SOE	13936	0.573	0.495	0.000	1.000	1.000
MARKET	13936	7.846	1.834	2.870	7.930	10.923

Note: Table 2 reports the descriptive statistics for the key variables. The variables are defined in Table 1. All of the continuous variables have been winsorized at the 1% and 99% levels.

**Table 3:** Correlation matrix.

	1	2	3	4	5	6	7	8	9	10	11
1.EID	1										
2.INTERACT	0.371 ***	1									
3.SIZE	0.285 ***	0.128 ***	1								
4.ROA	−0.005	0.012	−0.016	1							
5.LEV	0.121 ***	−0.035 ***	0.461 ***	−0.295 ***	1						
6.BOARD	−0.031 ***	−0.032 ***	0.205 ***	0.045 ***	0.074 ***	1					
7.INDEP	−0.332 ***	−0.353 ***	0.128 ***	−0.074 ***	0.105 ***	−0.089 ***	1				
8.TOP1	−0.041 ***	−0.083 ***	0.218 ***	0.122 ***	0.028 ***	−0.027 ***	0.021 **	1			
9.INS	0.147 ***	0.019 **	0.436 ***	0.049 ***	0.245 ***	0.075 ***	0.073 ***	0.297 ***	1		
10.SOE	0.185 ***	−0.136 ***	−0.407 ***	0.086 ***	−0.227 ***	0.067 ***	0.012	−0.073 ***	−0.207 ***	1	
11.MARKET	0.010	0.166 ***	−0.068 ***	0.063 ***	−0.176 ***	0.139 ***	−0.188 ***	−0.017 **	−0.095 ***	0.121 ***	1

Note: This table presents Pearson correlation matrices of the main variables. The variables are defined in Table 1. All the continuous variables are winsorized at the 1% and 99% levels. N = 13,936. ** *p* < 0.05, *** *p* < 0.01.

**Table 4:** Effect of social media interaction on EID.

	EID	
Variables	(1)	(2)
INTERACT		0.236 ***
		(10.483)
SIZE	0.847 ***	0.756 ***
	(11.896)	(10.493)
ROA	−4.245 ***	−4.024 ***
	(−5.102)	(−4.867)
LEV	0.625 *	0.826 **
	(1.825)	(2.425)
BOARD	0.113 *	0.124 **
	(1.893)	(2.136)
INDEP	−0.536 ***	−0.542 ***
	(−3.245)	(−3.368)
TOP1	0.119	0.463
	(0.281)	(1.095)
INS	−0.201	−0.233
	(−0.808)	(−0.940)
SOE	−0.107	−0.173
	(−0.645)	(−1.038)
MARKET	0.125 ***	0.168 ***
	(−3.237)	(−4.280)
Year	Yes	Yes
Industry	Yes	Yes
Constant	−12.951 ***	−11.195 ***
	(−8.731)	(−7.430)
N	13936	13936
R^2^	0.205	0.222
Adjusted R^2^	0.204	0.221
F	32.784	45.724

Note: Table 4 presents the results of OLS regression analysis related to the effects of social media interaction on EID. The t-statistics reported in parentheses are based on standard errors clustered by firm. * *p* < 0.1, ** *p* < 0.05, *** *p* < 0.01.

**Table 5:** Results of robustness tests.

	RKS	EID
Variables	(1)	(2)
INTERACT	0.216 ***	
	(2.768)	
INTERACT2		0.116 ***
		(8.220)
SIZE	−0.178	1.022 ***
	(−0.451)	(8.023)
ROA	1.312	0.136 ***
	(0.684)	(4.934)
LEV	−1.218	0.100 ***
	(−1.137)	(14.852)
BOARD	0.397 **	−0.122 *
	(2.282)	(−1.799)
INDEP	−0.460	0.101
	(−1.266)	(0.663)
TOP1	0.521	−4.303 ***
	(0.216)	(−5.140)
INS	−0.003	0.003
	(−0.427)	(1.006)
SOE	−0.689	0.382
	(−0.810)	(1.422)
MARKET	0.158	0.222 **
	(0.424)	(2.538)
Constant	37.372 ***	−17.965 ***
	(4.050)	(−6.700)
Year	Yes	Yes
Industry	Yes	Yes
N	3398	13936
R^2^	0.221	0.354
Adjusted R^2^	0.217	0.354
F	14.953	93.992

Note: Table 5 reports the fixed-effect model regression results for the robustness tests using alternative measurement of core variables. The t-statistics reported in parentheses are based on standard errors clustered by firm. * *p* < 0.1. ** *p* < 0.05. *** *p* < 0.01.

**Table 6:** Results of alternative estimation approaches.

	(1)	(2)	(3)
Variables	FE	Tobit	System GMM
INTERACT	0.588 ***	0.804 ***	0.238 ***
	(17.939)	(40.035)	(2.686)
SIZE	1.795 ***	1.313 ***	3.386 ***
	(12.051)	(28.415)	(3.175)
ROA	−1.279	−0.844	6.229**
	(−1.166)	(−1.140)	(2.157)
LEV	−1.689 ***	−0.548 **	−10.432 *
	(−2.989)	(−2.092)	(−1.872)
BOARD	0.153 *	0.143 ***	2.201 *
	(1.705)	(3.188)	(1.841)
INDEP	0.195	0.709 ***	0.208
	(0.754)	(5.754)	(0.045)
TOP1	−5.372 ***	−0.060 **	−35.259 ***
	(−5.424)	(−2.190)	(−3.517)
INS	0.669 **	0.098	−4.782
	(2.080)	(0.461)	(−1.480)
SOE	0.091	−0.777 ***	−4.296 ***
	(0.354)	(−8.335)	(−4.343)
MARKET	0.319 ***	0.089 ***	0.419 **
	(3.534)	(3.563)	(2.188)
L.EID			0.522 ***
			(8.061)
Year	Yes	Yes	Yes
Industry	Yes	Yes	Yes
Constant	−34.564 ***	−22.712 ***	−68.738 **
	(−10.874)	(−24.082)	(−2.472)
N	13936	13936	11993
Adjusted R^2^	0.226	0.358	
AR(2) *p*-value			0.746
Hansen *p*-value			0.642

Note: Table 6 presents the results of using alternative estimation approaches. The t-statistics reported in parentheses are based on standard errors clustered by firm. * *p* < 0.1, ** *p* < 0.05, *** *p* < 0.01.

**Table 7:** Two-stage least squares (2SLS) regression results.

Variable	INTERACT	EID
	(1) First Stage	(2) Second Stage
NETZEN	0.071 ***	
	(2.870)	
PREINTEACT		1.122 ***
		(13.931)
SIZE	0.071 ***	0.708 ***
	(5.390)	(15.099)
ROA	−0.002	0.125 ***
	(−0.424)	(10.214)
LEV	−0.004 **	0.073 ***
	(−2.062)	(16.123)
BOARD	−0.008	0.292 ***
	(−0.672)	(7.763)
INDEP	−0.063 **	−0.320 ***
	(−2.040)	(−3.092)
TOP1	−0.577 ***	1.169 ***
	(−6.401)	(3.667)
INS	−0.000	0.005 **
	(−0.667)	(2.322)
DUALITY	−0.079 **	−0.232 **
	(−2.517)	(−2.235)
SOE	−0.072 **	0.094
	(−2.413)	(0.982)
MARKET	−0.043 ***	−0.299 ***
	(−4.344)	(−11.111)
Constant	0.071 ***	−12.471 ***
	(2.875)	(−13.451)
Year	Yes	Yes
Industry	Yes	Yes
R^2^	0.617	0.258
N	13936	13936

Note: Table 7 reports the 2SLS regression results. The t-statistics reported in parentheses are based on standard errors clustered by firm. ** *p* < 0.05, *** *p* < 0.01.

**Table 8:** Results of mechanism analysis.

	Dependent Variable: EID		
Variables	(1)	(2)	(3)
INTERACT	0.187 ***	0.342 ***	0.129 ***
	(4.985)	(8.453)	(3.912)
INTERACT*ANALYST	0.023 **		
	(2.512)		
INTERACT*DUALITY		−0.139 ***	
		(−3.024)	
INTERACT*SOE			0.152 ***
			(4.014)
SIZE	0.842 ***	0.841 ***	0.842 ***
	(11.987)	(11.994)	(21.814)
ROA	−0.214	−0.209	−0.210
	(−1.490)	(−1.425)	(−1.632)
LEV	0.102	0.108	0.108
	(0.871)	(0.933)	(0.950)
BOARD	0.170 ***	0.169 ***	0.169 ***
	(5.507)	(3.247)	(3.241)
INDEP	−0.459 ***	−0.463 ***	−0.468 ***
	(−3.388)	(−3.423)	(−5.671)
TOP1	0.136	0.112	0.134
	(0.319)	(0.261)	(0.555)
INS	−0.001	−0.001	−0.001
	(−0.509)	(−0.484)	(−0.705)
DUALITY	0.198	0.400 ***	0.199 **
	(1.523)	(2.821)	(2.386)
SOE	−0.184	−0.183	−0.393 ***
	(−1.087)	(−1.080)	(−3.905)
ANALYST	−0.357 ***	−0.321 ***	−0.324 ***
	(−7.419)	(−7.372)	(−11.508)
MARKET	−0.193 ***	−0.193 ***	−0.192 ***
	(−4.909)	(−4.915)	(−9.230)
Year	Yes	Yes	Yes
Industry	Yes	Yes	Yes
Constant	−12.642 ***	−12.813 ***	−12.557 ***
	(−8.348)	(−8.501)	(−15.648)
N	13936	13936	13936
Adjusted R^2^	0.122	0.122	0.123
F	39.991	41.223	108.137

Note: Table 8 presents the results of mechanism analysis. The t-statistics reported in parentheses are based on standard errors clustered by firm. ** *p* < 0.05. *** *p* < 0.01.

**Table 9:** Differential effects on EID hard information and EID soft information.

	EIDhard	EIDsoft
Variables	(1)	(2)
INTERACT	0.235 ***	0.001
	(11.112)	(0.060)
SIZE	1.037 ***	0.287 ***
	(15.112)	(10.679)
ROA	0.135	−0.198
	(0.168)	(−0.574)
LEV	0.044	−0.042
	(0.134)	(−0.356)
BOARD	0.161 ***	0.048 **
	(2.925)	(2.439)
INDBOARD	−0.700 ***	−0.135 **
	(−4.599)	(−2.481)
TOP1	0.079	0.144
	(0.196)	(0.925)
INS	0.453 *	0.149
	(1.852)	(1.470)
SOE	−0.372 ***	0.518 ***
	(−3.131)	(9.326)
MARKET	−0.196 ***	0.003
	(−5.601)	(0.241)
Year	Yes	Yes
Industry	Yes	Yes
Constant	−16.147 ***	−6.209 ***
	(−11.382)	(−11.299)
N	13936	13936
R^2^	0.178	0.620
Adjusted R^2^	0.177	0.620
F	76.203	231.437

Note: Table 9 reports the heterogeneous effects of social media interaction on EID soft disclosure and EID hard disclosure. The t-statistics reported in parentheses are based on standard errors clustered by firm. * *p* < 0.1. ** *p* < 0.05. *** *p* < 0.01.

**Table 10:** Considering the topic of social media interaction.

	Dependent Variable: EID	
Variables	(1)	(2)
HJPOST	0.781 ***	
	(6.927)	
NHJPOST		0.224 ***
		(2.924)
	[*p* value = 0.021]	
SIZE	1.794 ***	1.760 ***
	(15.497)	(15.117)
ROA	0.142	0.146
	(0.088)	(0.090)
LEV	−2.015 ***	−1.687 ***
	(−3.202)	(−2.659)
BOARD	0.194 *	0.216 **
	(1.837)	(2.038)
INDEP	−0.984 ***	−1.058 ***
	(−3.376)	(−3.622)
TOP1	−1.080	−0.694
	(−1.358)	(−0.868)
INS	0.863 *	1.046 **
	(1.747)	(2.124)
SOE	−0.228	−0.379 *
	(−1.093)	(−1.836)
MARKET	0.175 ***	0.180 ***
	(2.929)	(3.021)
Year	Yes	Yes
Industry	Yes	Yes
Constant	−25.938 ***	−26.423 ***
	(−10.580)	(−10.691)
N	11178	11178
R^2^	0.277	0.274
Adjusted R^2^	0.276	0.273
F	118.069	115.781

Note: Table 10 presents the OLS regression results for the effect of different types of social media interaction on EID. * *p* < 0.1, ** *p* < 0.05, *** *p* < 0.01.

## Data Availability

The data presented in this study are available on reasonable request from the corresponding author.
